# Effect of Sperm Ratio and Temperature on Fertilization and Early Larval Development of the Surf Clam *Mesodesma donacium* (Bivalvia:Mesodesmatidae)

**DOI:** 10.3390/ani12172192

**Published:** 2022-08-26

**Authors:** Piera Vásquez-Calderón, Alejandro Abarca, L. René Durán, Doris Oliva

**Affiliations:** 1 Centro de Investigación y Gestión de Recursos Naturales (CIGREN), Facultad de Ciencias, Universidad de Valparaíso, Av. Gran Bretaña 1111, Valparaíso 2360102, Chile; 2 Departamento de Acuicultura, Facultad de Ciencias del Mar, Universidad Católica del Norte, Larrondo 1281, Coquimbo 1781421, Chile; 3 Copulhue SpA, Conchas Blancas, Achao 5750000, Chile; 4 Instituto de Biología, Facultad de Ciencias, Universidad de Valparaíso, Av. Gran Bretaña 1111, Valparaíso 2306102, Chile

**Keywords:** macha, fertilization, embryonic development, larval development, temperature, Southeastern Pacific

## Abstract

**Simple Summary:**

The surf clam *Mesodesma donacium*, known in the Southeastern Pacific as “macha”, has experienced decreased availability for local consumption due to high levels of extraction for many years in Chile and the effect of El Niño Southern Oscillation (ENSO) in Perú. To compensate the population decline, the advancement of the development of technology for cultivation and commercial production is proposed. Therefore, the development of small-scale aquaculture with the participation of artisanal fishermen and small fish farmers is considered. In this work, progress is made in determination of the conditions for fertilization and the effect of temperature on early embryonic and larval development.

**Abstract:**

The effect of sperm ratio on fertilization was evaluated in five sperm:oocytes treatments (10:1, 50:1, 100:1, 500:1 and 1000:1), the effect of temperature on embryonic and larval development in three temperature treatments (13 °C, 16 °C and 19 °C) was recorded and the duration of each stage, the growth rate and survival rate were registered. The oocytes were spherical (67.5 ± 4.2 μm) with a defined nucleus. Spermatozoa had a circular head (2 μm) and a fusiform flagellum (12 μm). The 500:1 sperm:oocytes treatment presented the lowest % of unfertilized oocytes, and lysis was observed in the 1000:1 treatment. An inverse relationship was observed between temperature and the duration of the stages of embryonic development. At 16 °C, veliger D larvae were observed at 41 h 45′ pf (88 ± 13.0 μm). Umbonate larvae were obtained at day 16 in the 13 °C culture and at day 10 in the 16 °C and 19 °C treatment (140 μm). On day 16 of culture, advanced umbonate larvae with a well-defined stomach (235 μm) were observed. The larval growth rate was higher in the 19 °C treatment (3.6 μm day^−1^) than the 13 °C and 16 °C treatment (2, 2.2 μm day^−1^). The mortality was higher in the 19 °C treatment (91%). These results are an initial contribution towards the culture of *M. donacium* as part of small-scale aquaculture in South America.

## 1. Introduction

*Mesodesma donacium* (Lamarck, 1818) (Bivalvia:Mesodesmatidae), commonly referred to as “macha” in Chile and Perú, is an endemic surf clam species in the Chile–Perú Malacological Zoogeographic Province [1], originally distributed in South America from Sechura Bay in northern Perú (5° S) to the Inio River at the southern tip of Chiloé Island in Chile (43° S) [2,3]. Nevertheless, after the extraordinary El Niño Southern Oscillation (ENSO) event in 1982–1983, the distribution was restricted in the northern range to 14° S and driven further south by El Niño in 1997–1998 [4].

In Chile, surf clam fishery has recorded landings since 1945, with very intense extraction between 1980 and 1999, reaching landings of up to 17,122 t in 1989 and decreasing to only 855 t in 2020 [5]. In Perú, fishery collapsed because of the overexploitation and the effects of El Niño in 1997–1998; therefore, the government decreed a ban in 1999. A recent stock assessment study showed a population abundance of only 110 t in Arequipa (between 15°30′ and 17°14′ S) [6].

At present, there are no commercial cultures of clams or surf clams in the Southeastern Pacific [7]. In 2020, 98% (400,000 t) of Chilean bivalve production was based on the culture of the mussel *Mytilus chilensis*, and the spats were collected from the wild [5].

*M. donacium* is a bivalve with biological potential for cultivation and commercial production in Chile [7]. The exploitation of this resource has experienced a great increase in the last 40 years; however, estimates of fishing effort have been scarce [8]. This species has been considered as a relevant resource for the diversification of Chilean aquaculture [9]. Small-scale aquaculture (APE) is an alternative for sustainable development in shellfish production, enhanced by artisanal fishermen and small farmers and supporting local food production and contributing to food security and household income in rural areas. Among the main challenges faced by its producers is access to quality spat [10].

The surf clam is a dioecious species without sexual dimorphism and with external fertilization that lives buried in the sand in the breaking sector of exposed and protected sandy beaches up to a depth of 15 m [3,11,12]. It has an annual reproductive cycle, with periods of maturation in winter and spawning during spring and summer. The recovery period of the gonad occurs in autumn [13]. It is characterized by emitting its gametes to the environment in a partial and asynchronous way [14] during warm periods with high food availability [15].

One of the techniques used to obtain both female and male gametes in surf clam broodstock is stripping, a procedure in which the gametes are extracted by successive cuts in the gonad and washed with sterile seawater [16,17,18].

The success of fertilization depends on the sperm:oocyte ratio, where low concentrations of sperm reduce the probability of encounters with the oocyte, and a high concentration of sperm can increase the risk of polyspermy that generates an abnormal development of the embryos and affects the survival of larvae [19,20,21,22,23]. On the other hand, physical factors, such as temperature, shape the speed of development, growth rate and mortality of embryos and larvae and thus are some of the most relevant factors for the success of marine cultures [24,25].

Methodologies for broodstock conditioning, spawning induction, fertilization conditions and the development of the initial phases are fundamental for success in bivalve mollusc cultures and are especially important in species with high fishing pressure and strong decreases in their landings [26]. The development of technology for species with potential for aquaculture promotes the diversification of aquaculture of native species, and hatcheries play a fundamental role in the supply of spats and enhance small-scale farming (APE) [18,27].

Currently the cultivation of the surf clam has been developed at an experimental level [7,12,16], and technological gaps in the larval, postlarval and grow-out stages still need to be resolved. New basic information on the cultivation of the surf clam could validate it as a potential native species for aquaculture diversification. In the present work, the effect of (a) sperm concentration on the success of fertilization and (b) temperature on embryonic and larval development is evaluated; this as an initial contribution to the massive spat production of this surf clam.

## 2. Materials and Methods

Broodstock of *Mesodesma donacium* (*n* = 93) of 63.77 ± 2.82 mm valvar length (antero-posterior axis) was obtained from a population in Cucao (42°38′ S; 74°06′ W) located on Chiloé Island during the maturation period in austral winter (June 2018). The specimens were transported to the hatchery of Copulhue SpA, located on Quinchao Island, and maintained in laboratory conditions until spawning. This was performed in a close water flow system with filtered seawater at 10 μm at a temperature of 13.3 ± 2.3 °C. The broodstock was randomly divided into two 1000 L tanks. A 30 L tray was placed at the top of each tank, and a 10 cm layer of fine sand was added as a substrate to each tray. Specimens were fed daily with a diet based on *Isochrysis lutea* and *Chaetoceros muelleri* in a ratio of 1:1 with a final concentration of 250,000 mL^−1^ cells. A visual examination was performed every week to determine changes in the size of the gonads in terms of sexual maturation, as per [28].

For the experiments, both female and male gametes were obtained by the “stripping” method. Successive cuts with a scalpel were performed on the gonad with caution so as not to cut the digestive gland and to avoid contamination. Subsequently, the gametes were dragged with a wash bottle with seawater filtered at 1 μm and stored in a 250 mL beaker where they were hydrated for 15 min for observation and measurement through a trinocular optical microscope (AMSCOPE 40C-200 x, AmScope, Irvine, CA, USA) with graduated eyepiece equipped with a digital camera using IPCapture software 9.1 (IMT, Burnaby, BC, Canada). For the evaluation of the quality of the gametes, motility of sperm and shape and size of oocytes were observed. Oocytes were counted in a Sedgewick Rafter egg count chamber (1 mL) and sperm in a 0.1 mL Neubauer count chamber.

### 2.1. Effect of Sperm Ratio on Fertilization

To assess the effect of sperm concentration on fertilization, increasing proportions of sperm per oocyte were used. Five treatments were used in a ratio of 10:1, 50:1, 100:1, 500:1 and 1000:1 sperm:oocytes, respectively. Glass containers of 250 mL with 200 mL of seawater filtered at 1 μm with a concentration of 20 oocytes per ml were used as per Helm, Bourne and Lovatelli [17]. Each treatment considered three replicates that were kept in a thermoregulated bath at 12 °C. Twenty-four hours post fertilization (pf), 3 samples of 1 mL were taken for every replica in each treatment, and the number of fertilized and non-fertilized oocytes was determined by trinocular optical microscope.

### 2.2. Effect of Temperature on Embryonic and Larval Development

After obtaining and quantifying gametes, the fertilization process was performed in plastic containers of 1200 mL with 1000 mL of filtered seawater in thermoregulated baths with a sperm/oocyte ratio of 100:1. Three temperature treatments were used, a 13 °C (12.73 ± 1.23) (autumn–winter temperature), a 16 °C (16.03 ± 0.72) (spring–summer temperature) and, finally, a high temperature in the zone of 19 °C (18.91 ± 0.65). Embryonic development was observed from fertilization until the presence of D larvae, approximately 48 h post fertilization (pf), by taking 1 mL subsamples for each treatment. The observations were made every 15 min during the first 3 h and every 30 min until D larvae were obtained. The duration of each stage was determined when 50% of the organisms attained a certain stage. The stages of development were observed and photographed under an optical microscope.

The same temperature treatments used in embryonic development in triplicate were used for larval development (from D larvae to umbonate larvae). For larval culture, an initial density of 4 larvae per mL was used with the seawater filtered (1 μm) and water exchanged every other day. The larvae were maintained through mixed feeding with microalgae (flagellates and diatoms) at a concentration of 20,000 cel/mL^−1^ [18], and growth rate and survival were evaluated by taking subsamples of 1 mL.

### 2.3. Data Analysis and Statistical Tests

The normality of the data was determined by a Shapiro–Wilk test and the homoscedasticity by a Levene test. The fertilization data (%) were transformed to the arcsine value for subsequent analysis. A one-way analysis of variance was used to identify significant differences between fertilized gametes in the five sperm:oocyte treatments. A significance level of α = 0.05 was used for all statistical analyses.

## 3. Results

**Figure 1:**
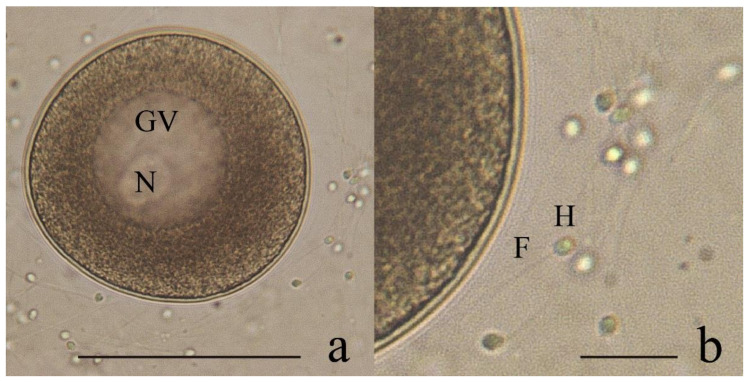
Oocyte and sperm of the surf clam *Mesodesma donacium* obtained by the stripping technique seen under a microscope. (**a**) Oocyte with germinal vesicle (GV) and nucleus (N), scale bar 50 μm; (**b**) sperm where the head (H) and flagellum (F) are observed (scale bar = 10 μm).

Of the gametes extracted using the stripping technique, an average of 322,528 ± 202,987 oocytes (*n* = 4) and 1,583,333 ± 418,911 spermatozoa (*n* = 3) was obtained. The female and male gonads did not differ in color or shape according to the naked eye, so microscopic analysis was required. The oocytes were spherical, with an average size of 67.5 ± 4.2 μm (*n* = 30), a defined nucleus and without a jelly coat. Spermatozoa were characterized by a rounded head (2 μm) and a fusiform flagellum (12 μm) (Figure 1).

### 3.1. Effect of Sperm Ratio on Fertilization Success

**Figure 2:**
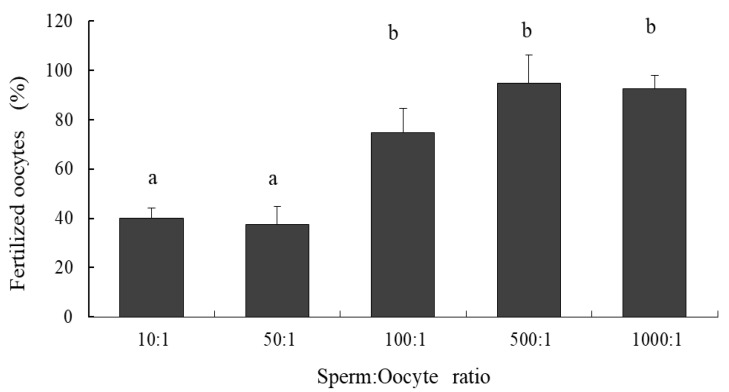
Fertilization success for the surf clam *Mesodesma donacium* using different sperm:oocyte ratios. Letters, **a** and **b**, indicate significative different groups (Tukey test). The vertical bar indicates SE.

The five sperm:oocyte ratio treatments showed significant differences in fertilization percentage (one-way ANOVA, df = 35, F = 27.75, *p* < 0.001). A Tukey’s a posteriori analysis (unequal N HSD) showed that the fertilization percentage ratios of 10:1 and 50:1 were significantly lower than the other sperm:oocyte ratios (≥100:1 sperm:oocyte ratio) (Figure 2). Treatments with the highest ratio of sperm per oocyte showed the lowest percentages of unfertilized oocytes. The presence of oocytes in lysis was observed in one replicate of the treatment with a ratio of 1000:1 sperm:oocyte.

### 3.2. Effect of Temperature on Embryonic and Larval Development

The embryonic development of *M. donacium* showed differences in the duration of the stages in the different temperature treatments. In general, an increase in temperature decreased the length of the different stages. Fecundation was characterized by the presence of a fertilization membrane with a diameter of 68 ± 4.5 μm at 15′ and 30′ pf in the treatment at 16 °C and 19 °C, respectively. At 13 °C, the rotatory blastula stage was observed at 10 h pf, while, at 16 °C and 19 °C, this phase was reached in 8 h 30′ and 7 h 30′ pf, respectively. Finally, type-D veliger larvae with a length of 88 ± 13.0 μm were obtained in the treatments at 13 °C and 16 °C. At 16 °C, the larval phase was achieved at 41 h 45′ pf and, at 13 °C, at 43 h 30′ pf (Table 1). The survival at the 14th day of culture was higher at 13 °C (22%) than at 16 °C (21%) and 19 °C (9%).

**Figure 3:**
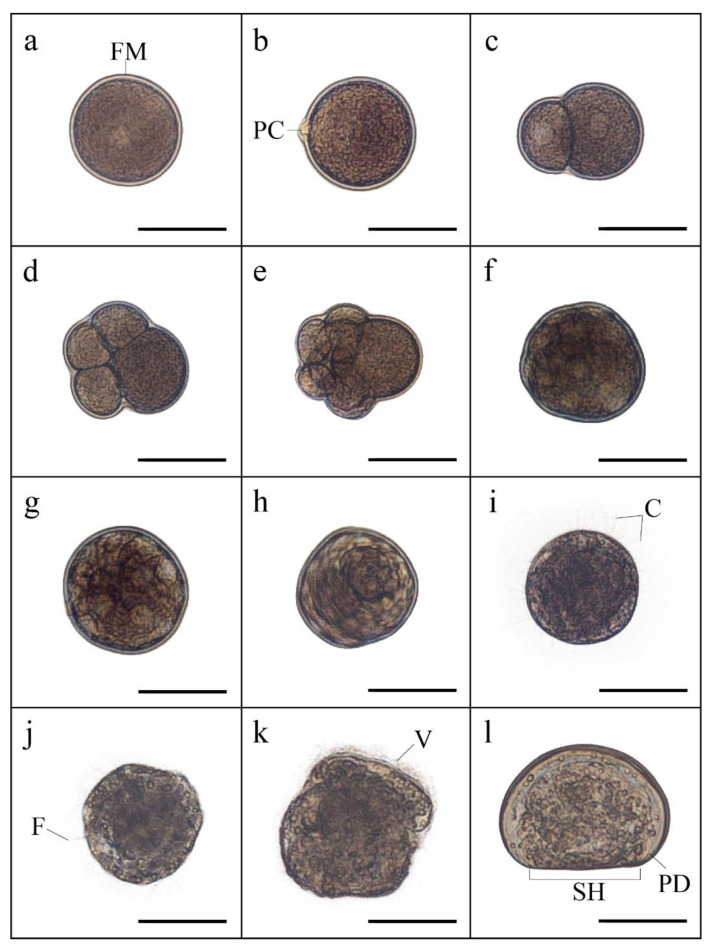
Embryonic development of the surf clam *Mesodesma donacium* at 16 °C: (**a**) oocyte with fertilization membrane (FM); (**b**) release of first polar corpuscle (PC); (**c**) first two-cell embryo segmentation (2 blastomeres); (**d**) second segmentation (4 cells); (**e**) third segmentation (8 cells); (**f**) morula; (**g**) blastula; (**h**) rotary blastula; (**i**) gastrula with cilia (C); (**j**) trochophore larva with apical flagellum (F); (**k**) trochophore larva with extended vellum (V); (**l**) D larva with straight hinge (SH) and prodisoconch I (PD). Scale bar = 50 μm.

The embryonic development was characterized by the presence of a fertilization membrane and, later, the presence of the first polar corpuscle, giving rise to successive segmentations until it reached the morula stage. Subsequently, there was a blastula during the first invagination, a rotating blastula with cilia around it, a gastrula in the second invagination, trochophore larva with the presence of a flagellum and great mobility until the formation of the early veliger larva stage with the presence of ciliated veil and, finally, veliger larva, type D, with a straight hinge and defined stomach (Figure 3).

**Figure 4:**
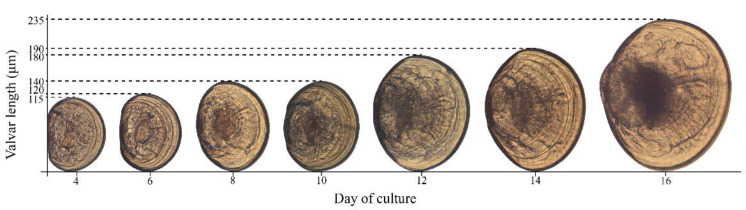
Larval development of the surf clam *Mesodesma donacium* maintained at 16 °C. The size of the larvae is shown from day 4 to day 16 post fertilization: D larva from day 4 to 10 and umbonate larva from day 12 to 16.

Temperature treatments showed differences in the duration of stages during larval development. Larval development from D larvae to umbonate larvae lasted 16 days at 13 °C but lasted 10 days at 16 °C and 19 °C (140 μm). In general, during day 6 and 8, no differences in the size or morphology of the larvae were observed. Later, on day 12 of culture, the umbonate larvae (180 μm) began to be observed in the treatment at 16 °C; on day 16 of culture, larvae with semi-curved hinges corresponding to advanced umbonate larvae with a well-defined stomach and a length of 235 μm (Figure 4) were observed. The larval growth rate was 2 μm day^−1^ at 13 °C, 2.2 μm day^−1^ at 16 °C and 3.6 μm day^−1^ at 19 °C.

## 4. Discussion

The “stripping” method [17] for obtaining gametes in *Mesodesma donacium* has been described as the fastest and most appropriate when mass fertilization is required [16]. The use of this technique has been applied by several researchers for this same species [11,12,29,30]. Of the gametes extracted by the “stripping” technique in this work, an average of 322,528 ± 202,987 oocytes and 1,583,333 ± 418,911 sperm cells was obtained. This number of oocytes was lower than that obtained by Illanes [31] using the same technique (3,330,401 ± 4,182,911 oocytes); the broodstock used by Illanes was collected in Caleta San Pedro (La Serena, Chile), with a total length and weight of 77.0 ± 4.7 mm and 45.3 + 7.2 g, respectively, unlike those used in this experiment, which measured 63.7 ± 2.8 mm and 25.0 ± 3.0 g. This could be explained by the differences in *M. donacium* population along the Chilean coast [32].

The oocytes obtained had an average diameter of 67.54 ± 4.2 μm, within the range of 65 to 70 μm, as described for the same species by Tarifeño [33], between 62 and 68 μm, as described by Zaro [12], and 63.5 ± 0.7 μm, as described Contreras [34] for specimens of *M. donacium.*

Regarding fertilization, in our experience, the ratio of ≥ 100:1 sperm:oocytes showed over 75% success in fecundation, and, in the 1000:1 sperm:oocytes treatment, oocytes in lysis were registered. Similarly, Le et al. [21] established an optimal ratio < 500:1 sperm:oocytes for *Panopea zelandica*, finding no significant differences in the percentage of embryos at 18 h pf between the ratio of 50:1 and 500:1; however, the ratio 50:1 showed a higher percentage (96%) of normal embryos. In our study, the result was the opposite, with greater fertilization obtained at higher sperm:oocyte ratios.

The triggers that modulate the reproductive cycle are both exogenous, such as temperature and food availability, and endogenous (genetic, hormonal, energy reserves) [35]. The diversity of environments and life habits of bivalves has promoted the development of different reproductive tactics, such as short or extended reproductive periods, adopting “r” or “k” reproductive strategies and producing a large or small number of offspring [36]. The satisfactory development of embryos under artificial conditions (hatchery) is essential for reliable larval production. The success of fertilization depends primarily on a good gametic ratio of sperm:oocytes, and this ratio depends on the endogenous and exogenous factors that regulate different processes in the reproductive cycle. Low sperm concentrations reduce the likelihood of oocyte encounters, and high sperm concentrations can increase the risk of polyspermy, which can lead to abnormal embryo development, affecting larval survival [19,20,21,23]. This optimal ratio varies between species of bivalves. In the clam *Clinocardium nuttallii*, an optimal sperm:oocyte ratio was found at a high value of 10,000:1 according to the results of Liu et al. [22]. In the *Spisula solidissima* clam, the best result was recorded in a range between 50:0 and 100:1 with 95% success in fertilization [23]. On the other hand, Dong et al. [19] observed an increase in the rate of polyspermy with a higher sperm:oocyte ratio and negative effects on larval survival. A concentration of < 200:1 was the most appropriate for the bivalve *Tegillarca granosa*. In Chilean clams with commercial value, the variation was very high between a ratio of 10:1 for *Gari solida* [37], a ratio of 100:1 for *Ensis macha* [38] and, finally, a ratio of 500:1 for *Tawera elliptica* [39].

It is difficult to make comparisons between different clams mainly due to the initial conditions of the broodstock at the beginning of the experiments and, thus, in the quality of the oocytes [40]. Embryogenesis and early larval development depend on the endogenous energy reserves supplied to the oocytes, and any energy deficiency can have serious consequences on subsequent success [41]. Cerviño-Otero [42] investigated the combined effect of seasonality and the geographical origin of broodstock on the number of eggs released and the larval yield of *Venerupis corrugata*, reporting significant differences in the biochemical composition of the gonads during the different seasons in all the studied populations.

It is important to note that the success of fertilization in bivalves is largely based on the sperm:oocyte ratio; however, there are other factors that can affect the production of D larvae. The density of the oocytes, the quality of the spermatozoa measured as motility, the contact time and the age of the gametes are also factors to be considered [20,43]. On the other hand, environmental factors, such as temperature, pH and salinity, also play an important role in the feasibility of restocking populations with a high distribution range [2,12,16,18,23]. In this study, the emission of gametes was through the “stripping” method, and comparisons with other species of clams where spontaneous spawning methods [22], application of desiccation stress and temperature [19,39] or injection of neurotransmitters [20,21,38] were used were difficult. Clotteau and Dubé [23] used the stripping method to obtain the gametes and required a high density of ml^−1^ oocytes (>10,000) and also a sperm:oocyte ratio > 100:1, which could be indicative that, depending on the type of spawning induction used [20,21], the process may require a greater or lesser ratio of sperm per oocyte and also different densities of oocytes.

Embryonic and larval development observed in *M. donacium* followed the patterns described for bivalve molluscs [7,17,37,39,44,45,46]. The effect of temperature on the first divisions, the stages of morula, blastula, gastrula and trochophore larva, was observed, with shorter developing times in higher-temperature treatments. The duration of the stages of development was similar to the results of Tarifeño [33] and Zaro [12]. They observed the trochophore larval stage at 24 h pf and veliger larva, type D, with a typical planktotrophic development of between 110 and about 250 μm, respectively, values similar to those obtained in this work (90 μm larva veliger, type D, and 235 μm umbonated 16-day-old larvae cultured at 16 °C). The diameter increased after the appearance of the fertilization membrane from 68 ± 4.5 μm to 88 ± 13.0 μm, and, after the formation of veliger larva, type D, the umbonate larva presented at 12 days pf in the treatment at 16 °C with a diameter of 140 μm, similar to that described for the family Mesodesmatidae (Table 2).

The larval growth observed in *M. donacium* suggests a direct increase in relation to temperature; the growth rate of the larvae (maximum valve length) was 2 μm day^−1^ at 13 °C, 2.2 μm day^−1^ at 16 °C and 3.6 μm day^−1^ at 19 °C. This value was lower than that obtained by Lépez [48] at 17 °C (10.52 μm day^−1^) but similar to that obtained by Guisado [52] with a valve growth of 3.3 μm day^−1^ in the same species. As mentioned, differences in larval culture results can be explained by the genetic origin, the sexual condition of the broodstock and the time of year, as well as the temperature and food availability. In this study, the broodstock was collected in austral winter (June 2018) when the breeders were in maturation stage. Reverol et al. [24] observed in *Paphies ventricosa* (Mesodesmatidae) that larval shell growth during the first 10 days was slow, with an increase in mean total length of 5.5 μm per day. On the other hand, Gadomski et al. [25] identified in *P. ventricosa* that the morphology of the larva does not depend on temperature; they only indicated a slowdown of larval development rather than physiological damage and abnormal development resulting from the increase in temperature, in the same way as observed in *Tivela mactroides*, where the increase in temperature promoted the accelerated development of the larvae with a greater growth rate. However, they pointed out that an increase in temperature could increase the possibility of occurrence of bacterial proliferations [24], which could be an explanation for the decrease in survival in higher temperature treatments in the present work.

Temperature accelerates metabolic processes, food activity and also growth in bivalve molluscs [17,53,54]. Temperature accelerates embryonic development in molluscs. Clotteau and Dubé [23] suggested that an adequate temperature range for early embryos in the clam *S. solidissima* is achieved between 15 °C and 20 °C. Lee and Rho [55] established that the time required to reach the stages of blastula, trochophora and D larva decreased to a third by increasing temperature from 8 °C to 17 °C in the *Panope japonica* clam. However, no survival data were presented. On the other hand, a lower temperature during the storage of the gametes before fertilization helped to extend the period in which the sperm remained motile, generating better management of the gametes in the hatchery [22,43].

In *M. donacium*, temperature generally decreases the duration of stages in embryonic development, increases growth and inversely influences survival. Zaro [12] determined that, above 21 °C, the larvae stage did not exceed 5 days and that, below 19 °C, the pediveliger larva stage was reached at 22 days with a 1.9% survival rate. Reverol et al. [24] observed that in *Tivela mactroides* the temperature (22 °C, 25 °C, 28 °C) was inversely proportional to the duration of the stages of larval development and directly proportional to survival rate. Albentosa et al. [53] mentioned that the general model of the effect of temperature can vary widely not only between species but within the same species. However, Reverol et al. [24] indicated that larvae, when approaching the juvenile stage, are more sensitive to temperature changes. In our experiment, *M. donacium* presented a high thermal sensitivity at the end of embryonic development at high temperatures (19 °C), with larvae after the stage of rotary blastula not being observed in that treatment: an important result highlighting the current climatic and environmental contingency.

*M. donacium* is a stenothermal species with a low tolerance to high temperatures related to its biogeographic origin and associated with the minimum oxygen zone and a high productivity associated with low temperatures in the upwelling ecosystem of the Humboldt Current [13,56]. Environmental phenomena, such as El Niño, which modifies large-scale environmental patterns in the Humboldt Current System, generate massive mortalities of species, which could affect the stability of the surf clam in the environment, especially in the meroplanktonic larval stages [57]. *M. donacium* also presented a high thermal sensitivity in the larval stages, mainly limiting growth.

It should be noted that the action of bacterial contamination or accumulation of materials and microorganisms (fibers, feces and protozoa) [24,58] are factors that affect larval survival and are enhanced by the increase in temperature in treatments. Reverol et al. [58] suggested that larval stages are especially sensitive to environmental changes, which could lead to larval mortality in the final phase of the cycle. Temperature influences survival and larval growth in bivalves; this factor is related to the ability to capture food (filterers), which modifies the filtration rate by factors such as temperature, the speed of water movement and the concentration of particles [24,59].

## 5. Conclusions

This study showed that the “stripping” method is effective for obtaining viable gametes in the surf clam. For the success of fertilization, it is recommended to use a ratio between ≥100:1 and ≤1000:1 sperm:oocytes. A temperature of 16 °C is suggested for the incubation of oocytes and for optimal embryonic and larval development for broodstock from the Cucao bank (Chiloé). The information obtained is fundamental for the development of a replicable culture technology that allows the scaling of larval crops of this species and optimizes the early stages of cultivation, enhancing it as an alternative for the sustainable development of raw materials with the support of small-scale aquaculture and contributing to food security and family income especially in rural areas. The main challenges to be addressed in the surf clam are the basic variables of larval culture such as larval density and feeding. It is also essential to study the phenomenon of metamorphosis in sandy substrate conditions and its subsequent cultivation in artificial and/or natural environments to finalize the cultivation technology of the species.

## Figures and Tables

**Table 1:** Time of developmental stages at different experimental temperatures (13 °C, 16 °C and 19 °C) from fertilized oocytes of the surf clam *Mesodesma donacium*.

	Temperatures			
Stages of Development	13 °C	16 °C	19 °C	Length (μm)
Fertilization membrane	30′	15′	15′	68 ± 4.5
First polar corpuscle	1 h 45′	1 h 30′	1 h 15′	69 ± 2.2
First segmentation	2 h 45′	2 h	1 h 45′	76 ± 5.5
Second segmentation	3 h 30′	2 h 30′	2 h 15′	*
Third segmentation or more	4 h 15′	3 h 15′	2 h 45′	*
Morula	5 h 30′	4 h 15′	4 h	74 ± 5.5
Blastula	6 h 45′	6 h	5 h 30′	*
Rotary blastula	10 h	8 h 30′	7 h 30′	*
Gastrula	12 h	9 h 15′	*	76 ± 5.5
Trocophora	26 h	23 h	*	78 ± 4.5
D larva	43 h 30′	41 h 45′	*	88 ± 13.0

* Not observed in the sample.

**Table 2:** Comparison of the size values of oocytes and larvae during the development of the surf clam *Mesodesma donacium* obtained in the present study with respect to results obtained with the same species and other species of the family Mesodesmatidae.

	Oocyte	Trochophore Larvae		D Larvae		Umbonate Larvae			
Species Common Name	Diameter (μm)	Length (μm)	pf	Length(μm)	pf	Length (μm)	pf	Temperature (°C)	Reference
*Mesodema donacium* Surf clam, macha	67.5 ± 4.2	78 ± 4.5	23 h	88 ± 13.0	43 h 30′	140	16 days	13	This study
			26 h		41 h 45′	140	10 days	16	
*M. donacium*	65–70	nd	24 h	nd	nd	nd	nd	nd	[33]
*M. donacium*	62–65	78.4± 3. 2	30 h	97.3 ± 4.3	45 h	129.1 ± 5.0	8	14.8 ± 0.6	[12]
*M. donacium*	63.5 ± 0.7	nd	nd	98.5 ± 1.5	48 h	nd	nd	16.7 ± 0.9	[34]
*M. donacium*	62–65	76.4 ± 3.2	24	93.8 ± 3.2	38 h	125.9 ± 4.7	6	19.2 ± 0.5	[12]
*M. donacium*	62–65	73,3 ± 3,7	24	84,2 ± 4,9	42 h	nd	nd	22.5 ± 0.6	[12]
*M. donacium*	50	nd	nd	92	26 h	205	nd	17	[47]
*M. donacium*	nd	nd	25 h	94 ± 5.5	44 h	188 ± 13	12 days	17	[48]
*M. donacium*	nd	nd	23 h	93–97	45 h	nd	8–12 days	18	[16]
*Mesodesma mactroides* Yellow clam	51.2 ± 6.6	57,86	18	79,69	24 h	83,07	8 days	25 ± 1	[49]
*Paphies ventricosa* Toheroa	60–66	nd	nd	85–105	24-48 h	109–320	nd	25	[46]
*P. ventricosa*			15 h		22 h		21	12	
	60.1–75.1	83–102	15 h	110–120	22 h	nd	15	16	[25]
			nd		37 h		12 days	20	
*Paphies subtriangulata* Tuatua	56-61	nd		88–143	24–48	125–265	nd	20	[50]
*P. subtriangulata*	56,3	56,81		96,94	24–36	134,51	6 days	22 ± 1	[51]

nd = no data.

## Data Availability

Not applicable.
